# Multiple susceptibility enhancer variants increasing *ADD3* expression predisposes to biliary atresia risk

**DOI:** 10.3389/fgene.2025.1733215

**Published:** 2026-01-12

**Authors:** Xinru Han, Haoyue Pei, Meirong Bai, Ying Zhou, Xun Chu

**Affiliations:** 1 Department of Pediatric Surgery, Xinhua Hospital Affiliated to Shanghai Jiaotong University School of Medicine, Shanghai, China; 2 Shanghai Institute of Pediatric Research, Shanghai, China; 3 Shanghai Key Laboratory of Pediatric Gastroenterology and Nutrition, Shanghai, China

**Keywords:** ADD3, biliary atresia, enhancer, SNP, zebrafish

## Abstract

**Background:**

Non-syndromic biliary atresia (BA) is a multifactorial disorder with a poorly understood genetic basis. We previously identified 154 BA-associated SNPs spanning the *ADD3* locus, which harbors the most strongly associated common variants in Asian populations. The susceptibility SNPs are in high linkage disequilibrium, but the causal variants remain unidentified.

**Methods:**

Using available databases, we predicted the regulatory potential of the 154 BA-associated SNPs. To functionally validate these findings, we assessed the enhancer activity of *cis*-regulatory elements (CREs) containing the risk variants using a dual-luciferase reporter assay. The role of *ADD3* dysregulation in hepatobiliary development was investigated using zebrafish. The spatiotemporal expression pattern of the *ADD3* ortholog in zebrafish embryos was mapped by *in situ* hybridization. Additionally, we performed mRNA overexpression and morpholino knockdown to examine the effects of perturbing *ADD3* ortholog expression on zebrafish hepatobiliary development.

**Results:**

Among 154 associated SNPs, 28 clustered within 10 putative CREs with predicted enhancer function. *In vitro* allele-specific luciferase assays demonstrated enhancer activity in eight of these CREs, with risk haplotypes at three loci driving significantly higher activity than non-risk haplotypes (*P <* 0.05). The zebrafish *add3a* gene, an ortholog of human *ADD3*, was expressed in developing livers. Both overexpression and knockdown of *add3a* in zebrafish disrupted hepatobiliary function and development, resulting in gallbladder hypoplasia/agenesis and reduced intrahepatic bile duct density. These phenotypes closely recapitulate BA pathology observed in humans. Combined with our prior data linking risk alleles to heightened *ADD3* expression and demonstrating *ADD3* overexpression in BA livers, these results indicate that genetic variants drive ADD3 upregulation, thereby predisposing to BA development.

**Conclusion:**

Multiple risk variants within enhancers upregulated *ADD3* expression, which contributed to BA pathogenesis.

## 1 Introduction

Biliary atresia (BA) is a rare cholangiopathy that occurs in infants (Nizery et al., 2016). The incidence of BA varies between 1 in 10,000 in the US and 3.7 in 10,000 in parts of Asia (Hartley et al., 2009; Chiu et al., 2013; Nizery et al., 2016). Biliary atresia (BA) exists in two distinct forms: syndromic BA (∼10%) and non-syndromic BA (∼90%). Syndromic BA is characterized by the presence of various congenital anomalies, such as polysplenia, asplenia, heterotaxy syndrome, intestinal malrotation, and an interrupted inferior vena cava (IVC). This form is typically associated with Mendelian inheritance patterns. In contrast, non-syndromic BA presents as isolated atresia of the bile ducts without additional congenital abnormalities. It is considered a multifactorial disease, influenced by both genetic and environmental factors (Chu et al., 2012; Asai et al., 2015; Bezerra et al., 2018; Malik et al., 2020).

BA is a progressive fibroinflammatory disorder of infants involving the extrahepatic and intrahepatic bile ducts (Matthews et al., 2011; Cui et al., 2013; Tam et al., 2024). The diagnostic hallmark of BA lies in extrahepatic duct anomalies, yet the disease’s prognosis is predominantly governed by the secondary progressive intrahepatic cholangiopathy that develops even after successful portoenterostomy (Matthews et al., 2011). Recent genetic discoveries implicate primary cilia as central to the pathogenesis of BA (Berauer et al., 2019; Lam et al., 2021; Glessner et al., 2023; Lim et al., 2024), and structural defects in primary cilia of cholangiocytes throughout both intra- and extrahepatic bile ducts in BA liver tissues (Frassetto et al., 2018). The human cholangiocyte H69 cell line has been extensively used as a human *in vitro* model to study the pathogenic mechanisms involved in human BA (Coots et al., 2012; Clemente et al., 2015; Zhao et al., 2020). Zebrafish (*Danio rerio*) exhibit remarkable evolutionary conservation in hepatobiliary morphogenesis and organogenesis with mammals. The extrahepatic biliary anatomy in zebrafish exhibits high conservation with mammalian systems, including the presence of a gallbladder. By 5 days post-fertilization (dpf), the zebrafish liver demonstrates well-differentiated hepatocytes and cholangiocytes, along with an established intrahepatic ductal network. Zebrafish has been extensively utilized as an animal model in BA research (Matthews et al., 2011; Cui et al., 2013; Glessner et al., 2023).

Genome-wide association studies (GWAS) studies and following replication studies have revealed rs17095355 in the Adducin 3 (*ADD3*) gene region as the most strongly associated common variant with BA susceptibility in Chinese populations (Garcia-Barceló et al., 2010; Wang et al., 2018; Bai et al., 2020; Cui et al., 2023). This association was also validated in Caucasians (Tsai et al., 2014). Our previous GWAS study and imputation analysis identified rs17095355 and other 153 SNPs within this genomic region exhibiting genome-wide significant associations with BA in 336 nonsyndromic BA infants and 8,900 controls. All these 154 SNPs were in high LD (r^2^ > 0.9) with rs17095355 (Cui et al., 2023). Furtherly, we found risk allele of rs17095355 was correlated with increased *ADD3* expression using eQTL analysis, and ADD3 was aberrantly deposit in the cholangiocytes and hepatocytes. Morpholino-mediated knockdown of *add3a* in zebrafish models resulted in hepatobiliary duct defects (Tang et al., 2016). However, the consequences of *ADD3* overexpression in hepatobiliary remain to be elucidated.

It is now widely recognized that most human complex diseases result from the cumulative genetic effects of hundreds to thousands of variants scattered throughout the genome (Visscher et al., 2017). At each susceptibility locus, multiple variants show statistically significant associations with the phenotype, but it is a challenge to clarify the number of functionally independent contributors. This challenge arises because genetic variants in close proximity are often correlated due to linkage disequilibrium (LD), which reflects shared inheritance patterns rather than shared biological effects. As a result, statistical methods alone cannot reliably distinguish causal variants from nonfunctional ones that are merely correlated through LD. Therefore, experimental validation by perturbing candidate variants and assessing their phenotypic impact is essential to resolve true causal relationships (Chatterjee et al., 2016; Chatterjee et al., 2021).

The *ADD3* variation was identified as the most strongly associated BA susceptibility locus. However, several major questions remain unanswered. First, because multiple SNPs in high LD were associated with BA risk, which SNPs affect *ADD3* expression? Second, do these disease-associated alleles increase BA susceptibility by upregulating or downregulating *ADD3* expression? Third, does upregulation of *ADD3* impair the structure and function of hepatobiliary system? In the current study, we searched the available databases to predict the potential functional consequences for the 154 associated SNPs. Then, we evaluated the allele specific expression of the candidate SNPs using dual-luciferase reporter assays. Finally, the zebrafish model was used to assess the impact of knockdown and overexpression of *ADD3* ortholog on the development of hepatobiliary duct and gallbladder.

## 2 Materials and methods

### 2.1 Screen for cis-regulatory element variants

We interrogated the 154 BA-associated SNPs against regulatory annotations from both ENCODE and HaploReg databases. SNPs overlapping histone marks, DNase I hypersensitive sites, protein binding regions, or transcription factor motifs were predicted to reside within cis-regulatory elements (CREs). We performed systematic screening of the SEdb 3.0 database to identify super enhancers that co-localize with experimentally validated enhancer-active CREs.

### 2.2 Cell culture

Human H69 cholangiocytes were cultured at 37 °C with 5% CO_2_ in Dulbecco’s modified Eagle’s medium (DMEM) supplemented with 10% fetal bovine serum (Gibco, California, United States) and 100 IU/mL penicillin (Gibco, California, United States).

### 2.3 Dual-luciferase report assay

DNA fragments flanking the 28 candidate SNPs were cloned into the pGL4.23 luciferase vector (Promega, United States). H69 cells in 24-well plates (Corning, United States) were co-transfected with either the pGL4.23 constructs or the empty pGL4.23 vector, along with the Renilla luciferase control vector, using Lipofectamine 8000 (Beyotime, Shanghai, China). Twenty-four hours post-transfection, luminescence was measured with the Dual-Luciferase Reporter Assay System (Yeasen, Shanghai, China) on a Synergy™ H1 Hybrid Multimode Microplate Reader (BioTek, United States). Firefly luciferase activity was normalized to Renilla luciferase activity for data analysis.

### 2.4 Zebrafish lines

All zebrafish experiments were performed using wild-type AB-strain reared at Xinhua Hospital. Procedures were conducted in accordance with institutional guidelines and approved by the Xinhua Hospital Animal Care and Use Committee (XHEC-WSJSW-2018-029).

### 2.5 Real-time polymerase chain reaction (RT-PCR)

Total RNA was extracted from 5 h post-fertilization (hpf) embryos using TRIzol reagent (Invitrogen, United States) in accordance with the manufacturer’s instructions. RT-PCR was performed using SYBR Green Master Mix with fluorescent labeling (Applied Biosystems, Cat#A25742, United States) on a QuantStudio Dx Real-Time PCR Instrument (Applied Biosystems, CA, United States). The 18S ribosomal RNA (18S rRNA) gene served as the normalization control. All assays were conducted in triplicate. Relative gene expression levels were calculated using the ΔΔ^CT^ method, expressed as RQ values (RQ = 2^−ΔΔCT^). Primer sequences used for RT-PCR are listed in (Supplementary Table S1).

### 2.6 Whole-mount in situ hybridization (WISH)

First-strand cDNA was then synthesized from 1 μg total RNA using PrimeScript RT Master Mix (Takara, Japan). PCR primers were designed to generate a 456-bp antisense riboprobe targeting zebrafish *add3a* (Supplementary Table S2). The PCR products were subsequently cloned into the pGEM-T Easy Vector (Promega, WI, United States). Digoxigenin-labeled antisense and sense RNA probes were then synthesized using the linearized plasmid templates (Roche Applied Science, Penzberg, Germany).

At 24 hpf, larvae were maintained in 0.003% 1-phenyl-2-thiourea (PTU; Sigma-Aldrich) to inhibit melanin formation. For fixation, zebrafish embryos were incubated overnight at 4 °C in 4% paraformaldehyde (Sangon, Shanghai, China), followed by dehydration and storage in 100% methanol (MeOH) at −20 °C for ≥2 h prior to processing. The chorions of larvae were manually removed under a stereomicroscope using fine forceps. Embryos >1 dpf were permeabilized with proteinase K (10 μg/mL) for 15–30 min (RT) to facilitate probe penetration.

Following prehybridization (2–4 h, 60 °C), embryos were hybridized overnight with 600 ng antisense probe at 60 °C. After blocking (3 h, RT), samples were incubated overnight at 4 °C with anti-DIG-AP antibody (1:5000; Roche). Signal detection was performed using BM Purple AP substrate (Roche) with subsequent imaging on a Nikon SMZ25 stereomicroscope (Chiyoda, Japan).

### 2.7 Morpholino antisense oligonucleotide design and injection

Morpholino antisense oligonucleotides (MOs) targeting *add3a* were designed and synthesized by GeneTools, LLC. A standard MO was used as the negative control. Translation-blocking (TMO) and splice-blocking (SMO) were designed to inhibit the 5′translational start site and a splice acceptor site respectively (Supplementary Table S3). Knockdown of *add3a* were mediated by injecting SMO and TMO into the yolk of 1 to 4-cell stage embryos. MOs were diluted to 0.25 mM in sterile double distilled water and approximate 2 nL/embryo MO was injected. To validate the efficiency of *add3a* splice-blocking morpholino (SMO), zebrafish embryos at 3 dpf were collected after microinjection. Total RNA was extracted, reverse-transcribed into cDNA, and analyzed by RT-PCR followed by gel electrophoresis to detect aberrant splicing products indicative of successful knockdown. To validate TMO efficacy, we PCR-amplified a 118-bp fragment encompassing the TMO target sequence from zebrafish cDNA and subcloned it into the pEGFP-N1 plasmid. Fluorescence attenuation following co-injection of TMO with this reporter construct confirmed successful translational inhibition. Primers are listed in Supplementary Table S4.

### 2.8 *In vitro* synthesis of mRNA and mRNA injection

Using cDNA from 5 dpf zebrafish eggs as the template, the full-length coding sequence of *add3a* was amplified by RT-PCR with the high-fidelity enzyme. Primers are listed in Supplementary Table S5. The full-length *add3a* fragment was purified by gel extraction. Homologous sequences corresponding to the linearized vector ends and restriction enzyme sites were added to the 5′ends of the primers. The pCS2+ empty vector was linearized by digestion with EcoR I. The purified PCR product was then cloned into the linearized pCS2+ vector in the SP6 forward orientation using the Hieff Clone Plus One Step Cloning Kit (YEASEN, China). The plasmid containing *add3a* was linearized with KpnI and used as a template for *in vitro* transcription to generate full-length mRNA. Capped mRNA was transcribed using the mMESSAGE mMACHINE Kit with SP6 polymerase (Thermo, United States). Embryonic microinjection 100 pg *add3a* mRNA was performed into one-to four-cell embryos.

### 2.9 PED6 treatment

PED6 is a fluorogenic substrate for phospholipase A2. It is metabolized in the liver and excreted into bile, accumulating in the gallbladder. To directly visualize the gallbladder, 5 dpf zebrafish embryos were incubated with 0.1 mg/mL PED-6 (Invitrogen, United States) for over 2 hours. Images from the PED-6 assay were acquired using a stereo fluorescence microscope (Leica, German) (So et al., 2018). Embryos in each group were categorized as “normal”, “faint”, or “absent” based on gallbladder size and the intensity of PED-6 fluorescence (Cui et al., 2013).

### 2.10 Biliary architecture analysis by whole-mount immunofluorescence

Zebrafish larvae at 5 dpf were fixed in methanol:DMSO (4:1) for 2 h at room temperature, followed by post-fixation in 100% ice-cold methanol for long-term storage. After rehydration through a graded methanol series in PBSX (PBS containing 0.5% Triton X-100), the larval epidermis was carefully removed to enhance antibody penetration. Samples were blocked for 1 h in 10% bovine serum albumin (BSA) with 0.5% Triton X-100 at room temperature.

Primary immunostaining was performed using mouse anti-Keratin 18 monoclonal antibody (Ks18.04; PROGEN, Cat#61028; 1:100 dilution in blocking buffer) overnight at 4 °C with gentle agitation. After extensive washing with PBSX, specimens were incubated with Alexa Fluor 488-conjugated goat anti-mouse IgG secondary antibody (YEASEN, Cat#33206ES60; 1:500 dilution) overnight at 4 °C.

Immunostained larvae were mounted in glycerol and imaged using a Leica TCS SP8 confocal microscope (Leica Microsystems, Germany). Images were processed using Adobe Photoshop CS6, and quantitative morphometric analyses (intrahepatic duct length and gallbladder area) were performed using ImageJ software (NIH).

### 2.11 Statistical analysis

Statistical analyses were performed using GraphPad Prism software. The data presented as mean ± SEM. Normality was assessed using the Shapiro-Wilk or Kolmogorov-Smirnov test (as appropriate), followed by Levene’s test for homogeneity of variance when parametric assumptions were met. Normally distributed data were analyzed using either a two-tailed Student’s t-test (for two-group comparisons) or one-way ANOVA (for multiple groups). The categorical data were analyzed using the χ^2^ test. A two-sided *P*-value < 0.05 was considered statistically significant for all analyses.

## 3 Results

### 3.1 Screen for cis-regulatory element variants

**FIGURE 1:**
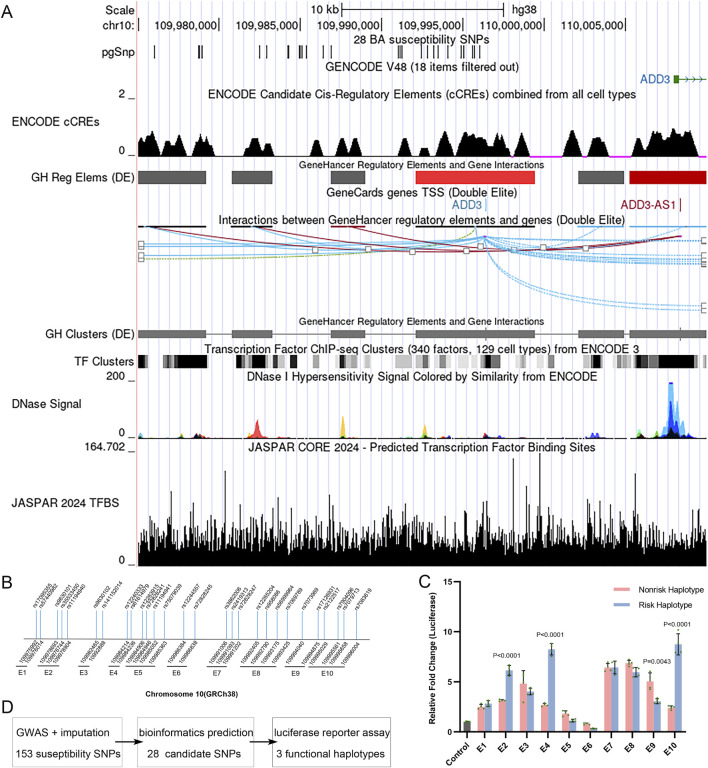
Genomic Map and Enhancer variants within the *ADD3* Locus. **(A)** Genomic locations of 28 BA-associated SNPs within predicted enhancer regions, marked by DNase I hypersensitivity, H3K4me1, and H3K27ac, and overlapping with predicted transcription factor binding sites. **(B)** Distribution of 28 BA-associated SNPs across 10 predicted enhancer segments. **(C)** Allele-specific luciferase reporter assays for 10 enhance elements. Eight elements demonstrated enhancer activity compared to promoter-only control, with four showing significant allelic differences (t-test). Error bars represent standard deviation (SD) of four biological replicates. **(D)** Flowchart outlining the approach for identifying causal variants among 154 BA-associated SNPs.

In a GWAS of BA susceptibility in Chinese individuals, we identified 154 SNPs reaching genome-wide significance (*P* < 5 × 10^−8^), including rs17095355 as the lead variant. All of these SNPs lie in the 5′upstream intergenic region or introns of *ADD3* and are in high LD with rs17095355 (r^2^ > 0.9). eQTL analysis revealed that the risk allele of rs17095355 correlated with increased *ADD3* expression. To identify causal variants among the 154 BA-associated SNPs, we screened for SNPs located within cis-regulatory elements (CREs) by querying data from ENCODE and HaploReg. This analysis revealed 28 SNPs across 10 distinct CREs (Figure 1), with spatially clustered SNPs co-localizing within the same CREs (Figure 1A). Among the 28 associated SNPs, 20 were located in regions containing histone marks characteristic for enhancers, 19 resided in DNaseI hypersensitive sites, seven overlapped transcription factor binding sites, and 24 were predicted to alter transcription factor motifs (Supplementary Tables S6, S7).

### 3.2 Functional tests of enhancer activity

We conducted *in vitro* functional tests of enhancer activity for the 10 CREs harboring 28 susceptibility SNPs. We cloned DNA fragments centered on the variants into the PGL4.23 luciferase vector and transfected them into Human H69 cholangiocytes (Table 1; Figure 1A). Totally, 10 elements were tested (Figure 1B; Table 1). Our results revealed that eight elements (E1, E2, E3, E4, E7, E8, E9 and E10) demonstrated significant enhancer activity (*P* < 0.0001 and > 2-fold increase in reporter activity compared to promoter-only control vector) and four elements (E2, E4, E9 and E10) showed allele-specific reporter activity differences (Figure 1C). Among the latter, 75% of risk haplotypes (E2, E4 and E10) exhibited higher enhancer activity than their non-risk counterparts (Figure 1D). Although the E1 region containing lead SNP rs17095355 showed strong enhancer activity in our assays, the similar activity levels between risk and non-risk haplotypes (*P* = 0.13) imply that this SNP might not be the functional variant responsible for the observed genetic association.

**TABLE 1:** 28 BA-associated SNPs located within 10 genomic elements with predicted enhancer function.

Genomic elements	Start	End	Fragments lengths (bp)	SNPs	Non-risk haplotype	Risk haplotype
E1	109975641	109976391	751	rs17095355- rs57440982	CG	TA
**E2**	**109978592**	**109979053**	462	**rs9630101-rs35533450-rs9630102**	**TGA**	**CdelT**
E3	109982314	109983064	751	rs11194940-rs141152014	GCA	AC
**E4**	**109984013**	**109984463**	451	**rs12240333-rs61614979**	**TA**	**AG**
E5	109984755	109985555	801	rs12263915-rs72828241-rs11194941-rs75079039	GTAT	CAGC
E6	109986233	109987083	851	rs12244557-rs72828245	TG	CT
E7	109990855	109991405	551	rs3862006-rs2419313-rs72828247	GGG	AAT
E8	109992254	109993604	1,351	rs12265204-rs958086-rs56999964-rs7069789	TGTC	CTCT
E9	109993914	109995064	1,151	rs7073969-rs17126931	CT	GC
**E10**	**109994928**	**109996128**	1,201	**rs2122517-rs7904096-rs7079713-rs7083619**	**CCCC**	**TTTT**

^a^ Del, deletion; rs35533450, G/deletion.

Bold elements (E2, E4, E10) exhibit enhancer activity, with risk haplotypes showing significantly stronger activity than non-risk haplotypes (luciferase reporter assays).

Super-enhancers (SEs) are clusters composed of multiple transcriptional enhancers that coordinately regulate gene expression (Wang et al., 2022). In this region, eight elements exhibiting enhancer activity were identified, suggesting that the locus may function as a SE. To explore this further, we queried SEdb 3.0. The results indicated this region acts as a SE across a broad range of tissues and cell types, including the liver tissue (Supplementary Table S8) (Wang et al., 2022).

At E2, the risk haplotype CdeletionT (rs9630101-rs35533450-rs9630102) showed 6.1-fold increased luciferase activity compared to the control (basal promoter), and 3.1-fold higher activity compared to the non-risk haplotype (*P* = 1.9 × 10^−5^). At E4, the risk haplotype TA (rs12240333-rs61614979) showed 8.2-fold increased luciferase activity compared to the control (basal promoter) and 2.7-fold higher activity compared to the non-risk haplotype (*P* = 1.4 × 10^−6^). At E10, the risk haplotype TTTT (rs2122517-rs7904096-rs7079713-rs7083619) showed 8.7-fold increased luciferase activity compared to the control (basal promoter) and 2.4-fold higher activity compared to the non-risk haplotype (*P* = 2.3 × 10^−5^). Our previous study found that the risk alleles were associated with increased expression of *ADD3*, which is overexpressed in BA cholangiocytes and hepatocytes (Cui et al., 2023). Taken together with the present results, these findings suggest that BA-associated risk alleles contributed to BA pathogenesis by upregulating *ADD3* expression.

### 3.3 Spatiotemporal expression of *add3a* in developing zebrafish embryos

**FIGURE 2:**
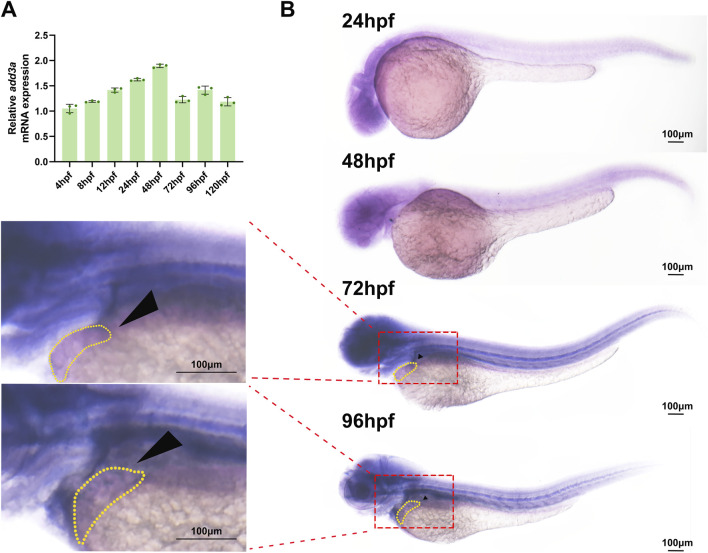
Spatiotemporal expression patterns of *add3a* in zebrafish embryos. **(A)** Relative mRNA level of *add3a* in zebrafsih embryos at 4, 8, 12, 24, 48, 72, 96 and 120 hpf; The data are shown as the mean ± SEM. **(B)** Spatial expression patterns of *add3a* was detected by WISH at 24, 48, 72 and 96 hpf zebrafish embryos. Black arrowheads marked livers. Scalebar = 100 μm hpf, hour postfertilization; SEM, Standard error of the mean; WISH, whole-mount insituhybridization.

To explore the role of *ADD3* dysregulation in hepatobiliary development, we employed zebrafish as a model system. We firstly used RT-PCR and WISH experiments to instigate whether *add3a*, the ortholog of human *ADD3* in zebrafish, was expressed in the developing liver of zebrafish. The qPCR results showed that the expression of *add3a* was present at 4 hpf, gradually increases until it peaks at 48 hpf, after which its expression slightly decreases and stabilizes (Figure 2A). The WISH results showed that *add3a* was mainly expressed in the head at 24-96 hpf, and was observed to be expressed in the liver at 72 hpf and 96 hpf (Figure 2B). These observed expression patterns strongly indicate the potential involvement of *add3a* in the development of hepatobiliary system.

### 3.4 Both overexpression and knockdown of *add3a* impair hepatobiliary development

Following our *add3a* expression analysis, we overexpressed *add3a* in zebrafish to model the upregulation effects observed in BA livers on hepatobiliary function and structure. Previous study reported that knockdown of *add3a* produced intrahepatic defects and impaired biliary function; therefore, we also performed a knockdown analysis for comparison (Tang et al., 2016).

**FIGURE 3:**
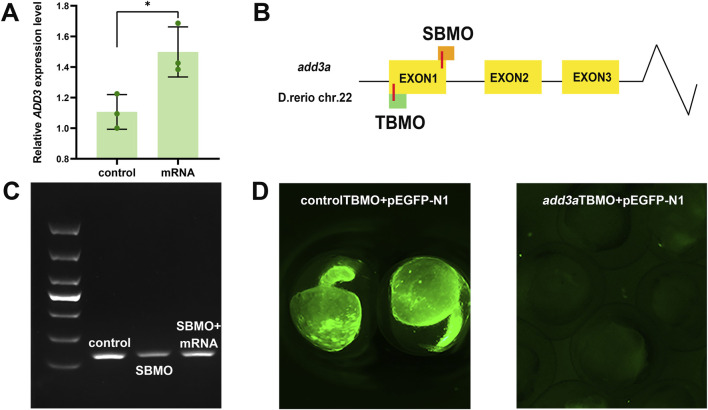
Validation of *add3a* upregulation via mRNA overexpression and knockdown using MOs. **(A)** RT-PCR confirmed successful overexpression of *add3a*, demonstrated by an increased level of *add3a* mRNA. **(B)** The target sites of *add3a*-SBMO and *add3a*-TBMO are shown. **(C)** RT-PCR and gel electrophoresis verified the effectiveness of *add3a*-SBMO, indicated by a decrease in band intensity. **(D)** The diminished fluorescence of PEGFP-N1 following co-injection with *add3a*-TBMO confirmed the effectiveness of *add3a*-TBMO (n = 20).

**FIGURE 4:**
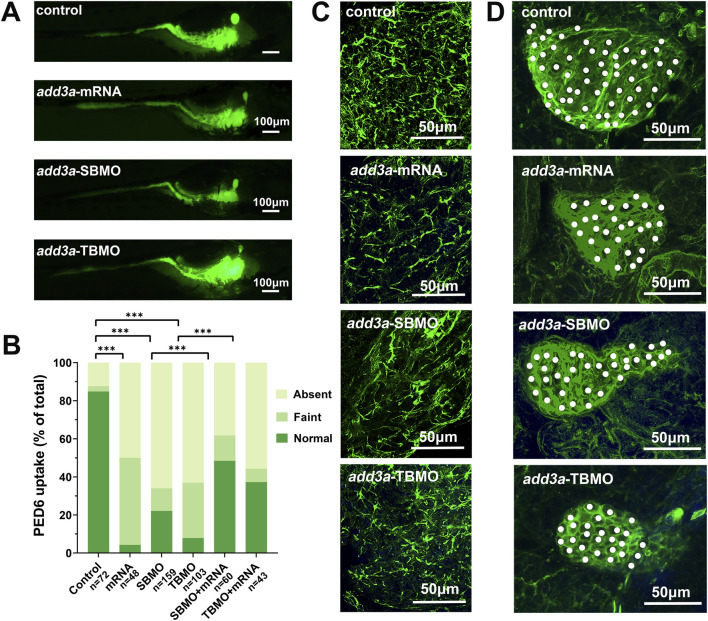
Knockdown or overexpression of *add3a* induces biliary dysfunction in zebrafish. **(A)** Lateral view of 5 dpf zebrafish larvae after ingestion of PED-6. The gallbladders of larvae injected with *add3a* mRNA or MOs were smaller compared to control embryos. **(B)** Quantitative analysis showed that the accumulation of PED-6 in the gallbladders of mRNA-injected larvae and morphants were significantly reduced compared to control larvae. Scale bar = 100 μm. Chi-square test was used. *P < 0.05; **P < 0.01; ***P < 0.001. **(C)** Confocal projection of 5 dpf larvae stained for cytokeratin 18, comparing control larvae, mRNA-injected larvae, and *add3a* morphants. The intrahepatic bile ducts in both mRNA-injected larvae and *add3a* morphants appeared sparser than in controls. n = 10 for each group. **(D)** Confocal projection of 5 dpf larvae stained for cytokeratin 18, showing that the gallbladders of both mRNA-injected larvae and *add3a* morphants were smaller than those of controls. n = 10 for each group. Gallbladder cells are indicated by white dots.

We overexpressed *add3a* by injecting its mRNA into zebrafish embryos to assess its effect on hepatobiliary development. RT-PCR showed a marked upregulation of *add3a* expression (Figure 3A). PED6 accumulation in the gallbladder was significantly reduced in *add3a* mRNA–injected larvae relative to controls (Figures 4A,B), implying impaired hepatobiliary function (Cui et al., 2013). We examined the liver and gallbladder by cytokeratin 18 immunofluorescence staining. Biliary abnormalities were evident in the *add3a*-overexpressing larvae compared with control larvae (Figures 4C,D). Relative to control MO larvae, *add3a*-overexpressing larvae exhibited reduced intrahepatic duct density and a decreased number of interconnecting ducts and terminal ductules (Figure 4C). Furthermore, the gallbladders were smaller, with significantly fewer cells and a smaller average cell area in the *add3a*-overexpressing larvae than in controls (Figure 4D).

We performed MO–mediated knockdown to reduce *add3a* expression, using a SBMO and a TBMO. The SBMO targets the exon 1 splice acceptor site of *add3a*, while the TBMO targets the translation start region in the first exon (Figure 3B). We confirmed MO efficacy: SBMO injection reduced band brightness on RT-PCR (Figure 3C), and TBMO injection abolished PEGFP-N1 plasmid fluorescence (Figure 3D). Co-injection of SBMO with overexpression constructs yielded higher band intensity than SBMO alone (Figure 3B). Rescue experiments with co-injection of full-length *add3a* overexpression mRNA alongside SBMO or TBMO partially restored the MO-induced phenotypes (Figure 4B), supporting MO specificity. SBMO- and TBMO-mediated knockdown reduced PED6 accumulation in the gallbladder and impaired intrahepatic ductal development as well as gallbladder morphology (Figures 4A–D).

Overall, our findings indicate that *add3a* is essential for normal hepatobiliary development in zebrafish. Hepatobiliary development is highly sensitive to *add3a* levels, as both overexpression and knockdown cause disruption, indicating precise dosage control is essential. Collectively, these *in vivo* data support the notion that BA-associated risk alleles contribute to BA pathogenesis by upregulating *ADD3* expression.

## 4 Discussion

Our previous study identified 154 non-coding SNPs in high LD associated with BA risk. In the current study, bioinformatics prediction revealed 28 SNPs lying in the enhancer region and disturbing transcriptional factors binding sites. Eight SNPs located in three regions showed enhancer activity and risk haplotypes exhibited higher enhancer activity than their non-risk counterparts. These results suggested multiple disease-associated SNPs might synergistically enhance ADD3 expression levels. Overexpression *ADD3* orthologue in zebrafish models caused impaired hepatobiliary function and structure, which mimic the phenotypes of BA in human.

Our current data from human genetics and *in vitro* functional tests of enhancer activity identified eight distinct CREs around *ADD3* with common sequence variants that are associated with BA. But only four CREs show allelic specific difference in enhancer activity, and the risk alleles of three showed upregulation activity. These CREs might control ADD3 expression within a topologically associating domain (TAD). It is reasonable to speculate that they act synergistically and cluster together as super-enhancers, thereby significantly amplifying *ADD3* expression. These findings align with observations from our prior study and other group, indicating that *ADD3* is over expressed in BA cholangiocytes and hepatocytes (Ye et al., 2017; Cui et al., 2023). Of note, the risk haplotype GC (rs7073969-rs17126931) at E9 showed 3.1-fold increased luciferase activity compared to the control (basal promoter), while 1.6-fold lower activity compared to the non-risk haplotype (*P* = 0.004). The risk haplotypes at three CREs (E2, E4, and E10) consistently demonstrated increased luciferase activity, whereas the risk haplotype at E9 showed reduced activity relative to its non-risk counterpart. This differential effect could potentially be explained by two non-exclusive mechanisms: (1) Tissue-specific regulatory context - The E9 locus may interact with biliary cell-specific repressive factors that are absent in our heterologous reporter assay system; and (2) Transcriptional resource competition - The GC risk haplotype might compete with other enhancer elements for limiting transcriptional machinery components, resulting in attenuated net activity. Taken together, the risk SNPs synergistically upregulate ADD3 expression, thereby contributing to BA pathogenesis.

**FIGURE 5:**
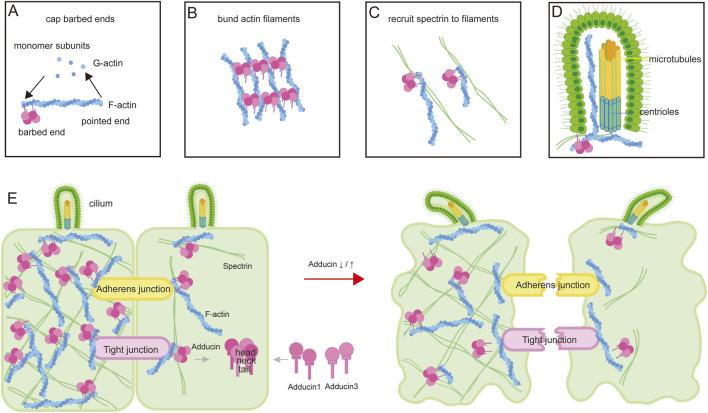
A schematic diagram illustrating the function of *ADD3* and the molecular mechanisms by which *ADD3* is involved in BA pathogenesis. **(A)** The actin cytoskeleton is composed of actin filaments polymerized from monomeric G-actin. Adducin, typically a heterodimer of ADD1 and ADD3, caps the barbed ends of actin filaments to inhibit further polymerization. **(B)** Adducins facilitate bundling of actin filaments. **(C)** Adducins recruit and crosslink spectrin at the filament termini, organizing the spectrin-actin network. **(D)** The cytoplasmic actin-adducin cytoskeleton regulates centrosome migration and docking to the apical plasma membrane, facilitates vesicle transport to the centriole, and mediates the entry of signal receptors into the ciliary membrane. Additionally, F-actin is a component of the axonemal cytoskeleton, positioned between alpha-tubulin singlets and located between microtubules and the ciliary membrane. **(E)** Adducins play a crucial role in maintaining cell shape, stabilizing junction complexes, and regulating ciliogenesis and ciliary function (left panel). Dysregulation of Adducin alters cell shape, impairs the stability of tight junctions and cell barrier integrity, disrupts cilia structure and function, and thereby contributes to BA pathogenesis (right panel).

*ADD3* encodes adducin 3. As depicted in Figure 5, adducins are tetrameric proteins composed of α (*ADD1*)/β (*ADD2*) or α/γ (*ADD3*) heterodimers. In most tissues, including the liver and biliary system, *ADD1* heterodimerizes with *ADD3*. As an actin-binding protein (Figures 5A,E), Adducin plays a crucial role in regulating filament dynamics by capping the fast-growing ends of actin filaments and facilitating their bundling (Figures 5A,B) (Moztarzadeh et al., 2022). Importantly, Adducin is a membrane-skeletal protein involved in the structural support of the cell membrane by linking the spectrin-actin network (Figure 5C). By stabilizing the connection between spectrin and actin, Adducin helps maintains cell shape and prevent mechanical instabilities in the cell membrane. Our results in zebrafish found that both knockdown and overexpression of *ADD3* orthologue resulted in abnormal gallbladder cells and sparse intrahepatic bile ducts. These findings implicate *ADD3* dysregulation results in altered cell shape and participates in the occurrence of BA (Figure 5E).

Spectrin-adducin-mediated membrane attachments of the perijunctional F-actin belt helps stabilize junctional structures by confining adherens junction (AJ)/tight junction (TJ) proteins at the apical cell surface and restricting their diffusion within the plasma membrane (Figure 5E) (Naydenov and Ivanov, 2011; Kugelmann et al., 2015). Depletion of adducin directly disrupts the formation of epithelial adherens junctions (AJs), which subsequently hinders the reassembly of tight junctions (TJs) (Moztarzadeh et al., 2022). Defects were observed in the assembly of cell junctions and polarity complexes in both cholangiocytes and hepatocytes in BA livers (Zhou et al., 2021; Amarachintha et al., 2022). These lines of evidence suggest *ADD3* dysregulation impair junction complex, thereby contributing to the pathogenesis of BA (Figure 5E).

Actin also plays a critical role in ciliogenesis and cilia maintenance in both the cytoplasm and the ciliary compartment (Hufft-Martinez et al., 2024). Cytoplasmic actin-adducin cytoskeleton controls centrosome migration and docking to the apical plasma membrane, vesicle transport to the centriole, and the entry of signal receptors within the ciliary membrane, thus regulating signaling (Figure 5D) (Francis et al., 2011; Yadav et al., 2016). F-actin also localize in primary cilia (Kiesel et al., 2020). It is a component of the axonemal cytoskeleton, and presents between alpha tubulin singlets and between the microtubules and ciliary membrane (Kiesel et al., 2020). It forms the site of ectocytosis, and regulates ciliary-mediated signaling. Actin is also found in the ciliary membrane, and thereby regulates ciliary membrane permeability (Zuo et al., 2019). Mutations in genes that affect the structure and function of cilia cause a group of inherited disorders called ciliopathies. Rare deleterious mutations are present in a broad range of liver-expressed ciliary genes in BA patients, implying BA as a type of ciliopathy (Lam et al., 2021). Since Adducin plays an important role in actin polymerization and cytoskeleton dynamic stability, we believe that ADD3 dysregulation directly impairs the ciliary homeostasis of cholangiocytes, subsequently influences BA pathogenesis (Figure 5E).

In conclusion, we identified multiple associated SNPs within *cis*-regulatory elements exhibiting enhancer activity. The risk alleles were associated with increased *ADD3* expression. Both upregulation and downregulation of *ADD3* disrupting hepatobiliary structure and function. The upregulation of *ADD3* driven by synergistic effects of these associated SNPs contributes to the pathogenesis of BA.

## Data Availability

The original contributions presented in the study are included in the article/Supplementary Material, further inquiries can be directed to the corresponding author.
