# Supplementary material for: Multiple susceptibility enhancer variants increasing ADD3 expression predisposes to biliary atresia risk
Source: Front Genet. 2026 Jan 12;16:1733215. doi: 10.3389/fgene.2025.1733215 (PMC12832116; doi:10.3389/fgene.2025.1733215)
Supplement: Supplementary file 1 [file Table1.docx]

**Supplementary Table 1. Primers for RT-PCR assays of *add3* expression levels in zebrafish larvae across developmental stages.**

| **Primer ID** | **Sequence** |
| --- | --- |
| *add3a* forward primer-103bp | 5’-GAAGAGAACCGACGCACCAA-3’ |
| *add3a* reverse primer-103bp | 5’-GAGGGACAAACTGGTTGGGA-3’ |
| *18s* forward primer-62bp | 5’-TCGCTAGTTGGCATCGTTTATG-3’ |
| *18s* reverse primer-62bp | 5’-CGGAGGTTCGAAGACGATCA-3’ |

RT-PCR: Real- time polymerase chain reaction; BP: base pair

**Supplementary Table 2. Primers for probe amplification used in Whole-mount In Situ Hybridization.**

| **Primer ID** | **Sequence** |
| --- | --- |
| *add3a* forward primer-456bp | 5’-ACCTGGGATACAGGACTGGC-3’ |
| *add3a* reverse primer-456bp | 5’-GAGGTCTCTCCACCACAATCC-3’ |

**Supplementary Table 3. MOs sequences.**

| **Primer ID** | **Sequence** |
| --- | --- |
| standard control MO | 5’- CCTCTTACCTCAGTTACAATTTATA -3’ |
| *add3a* splicing MO | 5’-GTGTGTGTTTCCTTACTGGACTC-3’ |
| *add3a* translation blocking MO | 5’-TCCTGTCTCGGCTCGCCACTCATCT-3’ |

MO: morpholino

**Supplementary Table 4. Primers for validating the efficiency of MOs.**

| **Primer ID** | **Sequence** |
| --- | --- |
| SMO forward primer-123bp | 5’-AAACACGCTGGCTCAACTCT-3’ |
| SMO reverse primer-123bp | 5’-CGCTAGCATTTCCCCCATCA-3’ |
| TMO forward primer-118bp | 5’-TAGAGATGAGTGGCGAGCC-3’ |
| TMO forward primer-118bp | 5’-GCGCGCAGATATTCAGG-3’ |

**Supplementary Table 5. Primers for full-length *add3a* cDNA amplification**

| **Primer ID** | **Sequence** |
| --- | --- |
| *add3a* forward primer-2112bp | 5’-GGATCCCATCGATTCGAATTCATGAGTGGCGAGCC  GAGACAGGAGGTGGTGACGAC -3’ |
| *add3a* reverse primer-2112bp | 5’-CTACTTGTCATCGTCATCTTTATAATCGTTGGAAA  AGGGCTTTATAAGTCCGGCTCGAGAGGCCTTGAATTCT TAGGCCTCCACTTTCTCTTTCTTTTTGTTCTTC-3’ |

**Supplementary Table 6. The allele frequency of 28 BA-associated SNPs with predicted enhancer function**

| SNP |  |  | Global | | East Asian | |
| --- | --- | --- | --- | --- | --- | --- |
|  | Ref Allele | Alt Allele | Ref Frequency | Alt Frequency | Ref Frequency | Alt Frequency |
| rs17095355 | C | T | 0.674 | 0.327 | 0.582 | 0.418 |
| rs57440982 | G | A | 0.685 | 0.315 | 0.588 | 0.412 |
| rs9630101 | T | C | 0.639 | 0.361 | 0.582 | 0.418 |
| rs35533450 | G | delG | 0.714 | 0.286 | 0.590 | 0.410 |
| rs9630102 | A | T | 0.640 | 0.360 | 0.582 | 0.418 |
| rs11194940 | G | A | 0.636 | 0.364 | 0.582 | 0.418 |
| rs141152014 | A | delA | 0.710 | 0.287 | 0.590 | 0.410 |
| rs12240333 | T | A | 0.671 | 0.330 | 0.581 | 0.419 |
| rs61614979 | A | G | 0.642 | 0.358 | 0.581 | 0.419 |
| rs12263915 | G | C | 0.671 | 0.330 | 0.581 | 0.419 |
| rs72828241 | T | A | 0.772 | 0.228 | 0.589 | 0.411 |
| rs11194941 | A | G | 0.671 | 0.330 | 0.581 | 0.419 |
| rs75079039 | T | C | 0.772 | 0.228 | 0.589 | 0.411 |
| rs12244557 | T | C | 0.670 | 0.330 | 0.581 | 0.419 |
| rs72828245 | G | T | 0.642 | 0.358 | 0.581 | 0.419 |
| rs3862006 | G | A | 0.692 | 0.308 | 0.592 | 0.408 |
| rs2419313 | G | A | 0.654 | 0.346 | 0.588 | 0.412 |
| rs72828247 | G | T | 0.712 | 0.288 | 0.546 | 0.454 |
| rs12265204 | T | C | 0.679 | 0.321 | 0.580 | 0.420 |
| rs958086 | G | T | 0.679 | 0.321 | 0.580 | 0.420 |
| rs56999964 | T | C | 0.661 | 0.339 | 0.580 | 0.420 |
| rs7069789 | C | T | 0.683 | 0.318 | 0.586 | 0.414 |
| rs7073969 | C | G | 0.680 | 0.227 | 0.580 | 0.411 |
| rs17126931 | T | C | 0.773 | 0.227 | 0.592 | 0.408 |
| rs2122517 | C | T | 0.548 | 0.453 | 0.583 | 0.417 |
| rs7904096 | C | T | 0.547 | 0.453 | 0.583 | 0.417 |
| rs7079713 | C | T | 0.551 | 0.449 | 0.583 | 0.417 |
| rs7083619 | C | T | 0.527 | 0.473 | 0.579 | 0.421 |

**Supplementary Table 7. Evidences supporting 28 BA-associated SNPs within 10 predicted enhancer regions**

| **SNP** | **Histone marks** | **DNaseI** | **proteins bound** | **Motifs change** |  |
| --- | --- | --- | --- | --- | --- |
|  |  |  |  |  |  |
|  |  |  |  |  |  |
| rs17095355 | yes (enhancer 11 tissues) | yes | no | 2 altered motifs |  |
| rs57440982 | yes (enhancer 11 tissues) | yes | no | 2 altered motifs |  |
| rs9630101 | yes (enhancer 14 tissues) | yes | 6 bound proteins | 3 altered motifs |  |
| rs35533450 | yes (enhancer 14 tissues) | yes | 1 bound protein | 1 altered motif |  |
| rs9630102 | yes (enhancer 12 tissues) | yes | 1 bound protein | 4 altered motifs |  |
| rs11194940 | yes (enhancer 13 tissues) | yes | 3 bound proteins | 2 altered motifs |  |
| rs141152014 | yes (enhancer 11 tissues) | yes | no | 3 altered motifs |  |
| rs12240333 | yes (enhancer 4 tissues) | yes | no | 1 altered motif |  |
| rs61614979 | yes (enhancer 4 tissues) | yes | no | 1 altered motif |  |
| rs12263915 | no | no | no | 2 altered motifs |  |
| rs72828241 | no | no | no | 14 altered motifs |  |
| rs11194941 | no | no | no | no |  |
| rs75079039 | no | no | no | 4 altered motifs |  |
| rs12244557 | no | no | no | no |  |
| rs72828245 | yes (enhancer 1 tissue) | no | no | 2 altered motifs |  |
| rs3862006 | no | no | no | 5 altered motifs |  |
| rs2419313 | no | yes | 1 bound protein | 2 altered motifs |  |
| rs72828247 | no | yes | 1 bound protein | 5 altered motifs |  |
| rs12265204 | yes (enhancer 7 tissues) | yes | no | 11 altered motifs |  |
| rs958086 | yes (enhancer 11 tissues) | yes | 1 bound protein | 13 altered motifs |  |
| rs56999964 | yes (enhancer 9 tissues) | no | no | no |  |
| rs7069789 | yes (enhancer 9 tissues) | no | no | 4 altered motifs |  |
| rs7073969 | yes (enhancer 6 tissues) | yes | no | no |  |
| rs17126931 | yes (enhancer 5 tissues) | yes | no | 6 altered motifs |  |
| rs2122517 | yes (enhancer 5 tissues) | yes | no | 5 altered motifs |  |
| rs7904096 | yes (enhancer 9 tissues) | yes | no | 2 altered motifs |  |
| rs7079713 | yes (enhancer 8 tissues) | yes | no | 1 altered motif |  |
| rs7083619 | yes (enhancer 8 tissues) | yes | no | 5 altered motifs |  |

**Supplementary Table 8. Super-enhancers located within the genomic region containing 28 BA-associated SNPs**

| **SE ID** | **SE Region** | **Tissue type** | **Biosample name** | **SE rank** | **Element** | **Common SNP** | **eQTL** | **Risk SNP** | **TFBS** | **CRISPR Cas9** | **Gene** |
| --- | --- | --- | --- | --- | --- | --- | --- | --- | --- | --- | --- |
| SE_02_041100186 | chr10:109955677-109980864 | Liver | TTC549_untreated | 186 | 4 | 629 | 119 | 5 | 171 | 0 | ADD3, XPNPEP1 |
| SE_02_041200201 | chr10:109956685-109981161 | Liver | TTC549_Doxycycline | 201 | 4 | 614 | 116 | 5 | 168 | 0 | ADD3, XPNPEP1 |
| SE_02_265700392 | chr10:109961123-110010527 | liver | Huh7 | 392 | 11 | 1304 | 294 | 4 | 60 | 309 | ADD3-AS1, ADD3, XPNPEP1 |
| SE_02_143800647 | chr10:109962695-110016678 | Liver | LSEC | 647 | 13 | 1438 | 327 | 4 | 257 | 326 | ADD3-AS1, ADD3, XPNPEP1 |
| SE_02_265500489 | chr10:109964617-110010909 | liver | Huh7_siNC | 489 | 13 | 1232 | 269 | 3 | 59 | 309 | ADD3-AS1, ADD3, XPNPEP1 |
| SE_02_143601184 | chr10:109965149-109997123 | Liver | LSEC-Ctrl | 1184 | 11 | 832 | 170 | 2 | 144 | 33 | ADD3-AS1, ADD3, XPNPEP1 |
| SE_02_260200304 | chr10:109965182-110003696 | liver | siNCOA3 | 304 | 18 | 1011 | 230 | 3 | 59 | 33 | ADD3-AS1, ADD3, XPNPEP1 |
| SE_02_260000412 | chr10:109965304-110012311 | liver | siNC | 412 | 10 | 1258 | 281 | 3 | 69 | 309 | ADD3-AS1, ADD3, XPNPEP1 |
| SE_01_003700225 | chr10:109966197-110009231 | Liver | hepatocyte | 225 | 8 | 1133 | 251 | 3 | 212 | 326 | ADD3, XPNPEP1 |
| SE_02_113300438 | chr10:109966584-110008735 | Liver | Hepatic stellate cells | 438 | 8 | 1098 | 240 | 3 | 204 | 326 | ADD3-AS1, ADD3, XPNPEP1 |
| SE_02_275700290 | chr10:109967552-110010986 | liver | Huh7.5_HCV | 290 | 14 | 1152 | 255 | 3 | 58 | 309 | ADD3-AS1, ADD3, XPNPEP1 |
| SE_02_271601082 | chr10:109995038-110011309 | liver | Huh7.5 | 1082 | 4 | 460 | 117 | 1 | 19 | 309 | ADD3-AS1, ADD3 |
| SE_02_034200980 | chr10:110058222-110087096 | Liver | HSPCs_F0 | 980 | 6 | 771 | 122 | 1 | 98 | 0 | ADD3 |

SE: Super enhancer
